# Sleep-Wake Evaluation from Whole-Night Non-Contact Audio Recordings of Breathing Sounds

**DOI:** 10.1371/journal.pone.0117382

**Published:** 2015-02-24

**Authors:** Eliran Dafna, Ariel Tarasiuk, Yaniv Zigel

**Affiliations:** 1 Department of Biomedical Engineering, Faculty of Engineering, Ben-Gurion University of the Negev, Beer–Sheva, Israel; 2 Sleep-Wake Disorders Unit, Soroka University Medical Center, and Department of Physiology, Faculty of Health Sciences, Ben-Gurion University of the Negev, Israel; The University of Science and Technology of China, CHINA

## Abstract

**Study Objectives:**

To develop and validate a novel non-contact system for whole-night sleep evaluation using breathing sounds analysis (BSA).

**Design:**

Whole-night breathing sounds (using ambient microphone) and polysomnography (PSG) were simultaneously collected at a sleep laboratory (mean recording time 7.1 hours). A set of acoustic features quantifying breathing pattern were developed to distinguish between sleep and wake epochs (30 sec segments). Epochs (n = 59,108 design study and n = 68,560 validation study) were classified using AdaBoost classifier and validated epoch-by-epoch for sensitivity, specificity, positive and negative predictive values, accuracy, and Cohen's kappa. Sleep quality parameters were calculated based on the sleep/wake classifications and compared with PSG for validity.

**Setting:**

University affiliated sleep-wake disorder center and biomedical signal processing laboratory.

**Patients:**

One hundred and fifty patients (age 54.0±14.8 years, BMI 31.6±5.5 kg/m2, m/f 97/53) referred for PSG were prospectively and consecutively recruited. The system was trained (design study) on 80 subjects; validation study was blindly performed on the additional 70 subjects.

**Measurements and Results:**

Epoch-by-epoch accuracy rate for the validation study was 83.3% with sensitivity of 92.2% (sleep as sleep), specificity of 56.6% (awake as awake), and Cohen's kappa of 0.508. Comparing sleep quality parameters of BSA and PSG demonstrate average error of sleep latency, total sleep time, wake after sleep onset, and sleep efficiency of 16.6 min, 35.8 min, and 29.6 min, and 8%, respectively.

**Conclusions:**

This study provides evidence that sleep-wake activity and sleep quality parameters can be reliably estimated solely using breathing sound analysis. This study highlights the potential of this innovative approach to measure sleep in research and clinical circumstances.

## Introduction

Polysomnography (PSG) is currently considered the gold standard for sleep evaluation [1]. This method requires a full night laboratory stay and subjects are connected to numerous electrodes and sensors, which are attached on the patient's body. Time series data are aggregated, processed, and visually examined or mathematically transformed in order to reveal insights about sleep-wake states and many aspects of physiology. Moreover, in routine sleep diagnostic procedures, sleep scoring is done manually by applying complex and visual scoring rules simultaneously on multiple signals acquired by applying contact sensors, e.g., electroencephalography (EEG), electrooculography (EOG), electromyography (EMG), electrocardiography (ECG), and respiratory activity [1,2]. PSG is time-consuming, tedious, and costly due to complexity and the need for technical expertise.

Currently, the biomedical engineering field of sleep disorders evaluation is on a “fast track” towards ambulatory sleep medicine [3–5]. In recent years, extensive effort has been devoted to seeking alternative simple cost-effective technologies for objective sleep-wake evaluation to increase accessibility in sleep disorders diagnosis. These new technologies are based on reduced-channels and sensors, and sophisticated computer-based algorithms [3,4,6–9]. Under the assumption that movement is associated with wake phase and lack of movement implies a sleep phase, clinicians and researchers have attempted to measure the binary presence of sleep or wake phases by measuring wrist movements using actigraphy [5,10,11]. Field-based activity monitoring devices are increasingly used as simple and cheap accelerometer-based devices [12–14]. Montgomery-Downs et al. [13] recently reported that this new technology has specificity limitations similar to those of a traditional actigraphy device. These devices consistently misidentify wake as sleep and thus overestimate both sleep time and quality.

It was long established that central control of ventilation and upper airway patency are strongly affected by transitions from sleep to wake and vice versa [4,15,16]. During sleep, there is a considerable increase of upper airway resistance [4,17,18] due to decreased activity of the pharyngeal dilator muscles [19,20]. This elevated resistance is reflected by amplification of air-pressure oscillations in the upper airways during breathing. These air-pressure oscillations are perceived as breathing sounds during sleep [21]. In contrast, during wakefulness, there is an increase in activity of the upper airway dilating muscles, hence decreased upper airway resistance and airway oscillations. Recently, we have shown that it is possible to accurately detect and distinguish a whole night’s breathing sounds and snoring events from environmental noises [22]. We also demonstrated that the audio signal can be acquired using a non-contact sensor (ambient microphone), which minimizes the interruption of sleep [22,23]. However, little is known about whether acoustic-breathing parameters can distinguish between sleep-wake patterns.

We hypothesize that sleep-wake activity can be estimated using audio signal analysis of breathing sounds, which are altered by changes in ventilation and upper airway patency. The objectives of our work are: 1) to develop a breathing sound analysis (BSA) algorithm for distinguishing between sleep and wake phases using non-contact technology; 2) to reliably estimate sleep quality parameters such as total sleep time, sleep latency, sleep efficiency, wake after sleep onset time, and arousal index; and 3) to validate the proposed algorithms in comparison to PSG.

## Methods

This article has online Supporting Information, S1 Dataset.

### Setting

University affiliated sleep-wake disorder center and biomedical signal processing laboratory. This study analyzed routinely collected data and the data were analyzed anonymously; therefore informed consent was not required. The Institutional Review Committee of Soroka University Medical Center approved this study protocol (protocol number 10141). The institutional review board waived the need for written informed consent from the participants.

### Subjects

We prospectively recruited 150 consecutive adults (aged 19 to 86 years, 53/97 female/male) referred to the Sleep-Wake Unit for routine PSG study for sleep disorders diagnosis, starting February 2008. We selected the first 80 subjects (patients) for the system design (training) study; the remaining 70 subjects (starting July 2010) were included in the blind validation study. See Table 1 and Table 2 for patient characteristics and sleep quality assessments.

**Table 1:** Subject anthropometric parameters.

Anthropometric parameters	System Design (n = 80)	System Validation (n = 70)	*p*
**Gender (M/F)**	54/26	43/27	.437
**Age (yr)**	52.8±13.1 (19–82)	55.4±16.8 (19–86)	.289
**BMI (kg/m^2^)**	31.8±5.0 (20.2–52.1)	31.4±6.0 (16.8–47.2)	.400
**ESS (score)**	9.7±6.0 (0–23)	10.3±5.9 (0–24)	.539
**AHI (events/hr)**	19.0±18.3 (0.4–76.7)	17.9±16.8 (0.0–84.1)	.703

BMI—body mass index, ESS—Epworth sleepiness scale, AHI—apnea hypopnea index. All values are mean ± SD (range). *p* value was calculated using unpaired t-test for age, BMI, ESS, and AHI; and chi square for gender.

**Table 2:** Sleep Quality Parameters.

Sleep quality parameter	PSG System Design (n = 80)	PSG System Validation (n = 70)	BSA System Validation (n = 70)	Difference (BSA-PSG)	Absolute Error
**TIB**	422.2±54.5	428.5±51.0	428.5±51.0	–	–
**(min)**	(285.0–503.0)	(339.0–496.0)	(339.0–496.0)		
**TST**	333.0±52.4	339.1±60.3	343.0±54.1	7.6±48.1	35.8±32.8
**(min)**	(187.0–411.0)	(148.5–432.0)	(151–499)	(-105.0–119.5)	(1–119.5)
**SL**	30.1±29.8	36.0±23.6	34.4±18.6	-3.3±23.6	16.6±16.9
**(min)**	(0.5–100.0)	(0.5–125.0)	(1–125)	(-59.0–67.0)	(0–67)
**SE**	79.8±12.9	76.1±13.5	76.8±11.3	1.5±10.7	8.0±7.3
**(%)**	(39.4–97.5)	(30.9–97.7)	(31–95)	(-26.7–27.1)	(0.2–27.1)
**WASO**	46.1±39.2	56.9±46.0	60.7±39.3	7.8±41.3	29.6±29.7
**(min)**	(3.0–179.0)	(7.0–253.0)	(12–244)	(-117–116)	(0–117)
**AwI**	1.4±1.1	1.2±0.9	1.3±0.7	0.2±1.0	0.8±0.8
**(events/hr)**	(0.0–6.2)	(0.0–2.8)	(0.1–2.8)	(-1.3–3.9)	(0–3.9)

TIB—Time in bed; PSG—Polysomnography; BSA—breathing sound analysis; TST—Total sleep time; SL—Sleep latency; SE—Sleep efficiency; WASO—Wake time after sleep onset; AwI—Awakening index. The differences between the sleep quality parameters as measured by PSG and BSA are presented to show the direction of any bias. Absolute error (difference) was presented to quantify the overall magnitude of differences among measurements. Values are mean ± SD (range) between subjects.

### PSG study

Subjects reported to the laboratory at 20:30 and were discharged at 06:00 the following morning. They were encouraged to maintain their usual daily routine and to avoid any caffeine and/or alcohol intake on the day of the study. The laboratory environment was sleep-friendly according to recommendations of the National Sleep Foundation [24].

PSG study (SomniPro 19 PSG, Deymed Diagnostic, Hronov, Czech Republic) included EEG (referential derivations, international 10–20 system, C3/A2, C4/A1, and O2/A1, O1/A2), EOG, EMG, ECG, respiratory activity (abdomen and chest effort belts—respiratory inductance plethysmography), oxygen saturation, and snore level intensity (Quest Technology 2700, Orlando, FL, USA). Simultaneous video monitoring was digitally recorded. PSG scoring includes sleep-wake pattern determined by a trained technician and underwent a second scoring by one of the investigators (AT); scoring followed the American Academy of Sleep Medicine criteria [1]. The scoring included labeling of each (30 sec) epoch as sleep or wake using the PSG signals—this was used as the gold-standard labeling for the training and validation of the proposed breathing sound analysis system. For labeled data visualization see Figure A in S1 Dataset.

### Study protocol

**Fig 1:**
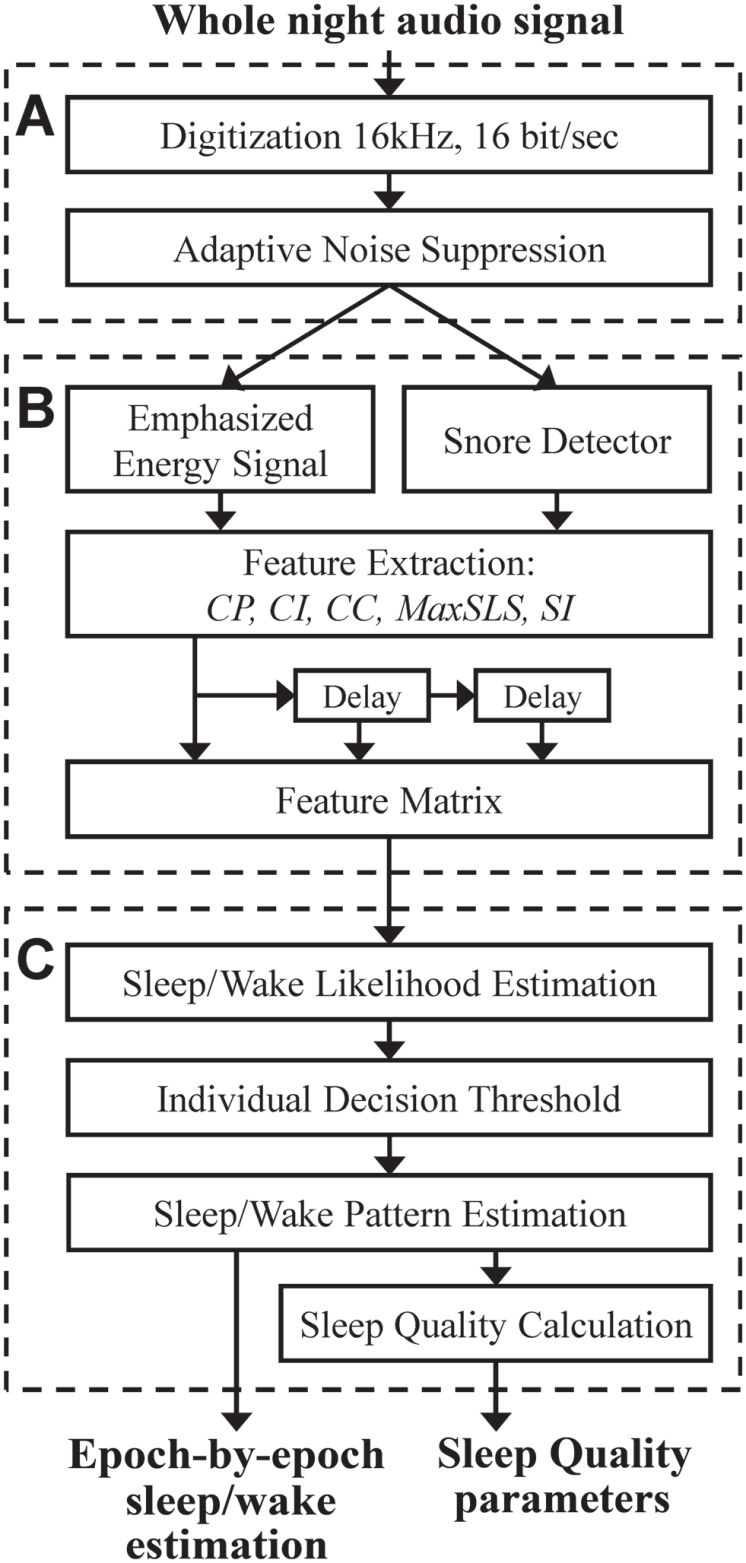
Block diagram of the proposed system. The system consists of three main stages: A) Preprocessing and signal enhancement. B) Feature extraction. C) Sleep/wake Estimation.

We developed a system for sleep-wake pattern estimation and sleep quality evaluation. The system is composed of a non-contact microphone, digital audio recording device, and an algorithm that estimates sleep and wake states from a full night audio recording (Fig. 1). Using concepts from audio signal processing and pattern recognition techniques, acoustic properties of breathing were classified into two states: sleep and wake. Breathing sounds events were automatically located, segmented, and isolated using our breathing detection system [22] that is capable of detecting even low intensity (>20 dB) breathing sounds. This system was validated to distinguish breathing sounds from irrelevant noises, such as movements, linen noises, speech, and other interferences. Using the detected breathing events and the calculated energy signal from the audio recordings, eight acoustic features were developed and extracted per subject; these features express the acoustic properties of breathing events and emphasize the differences between sleep and wake phases. These eight features were used for training an AdaBoost [25] classifier configured as a time-series model that aimed to classify each 30-sec epoch as sleep or awake. Finally, sleep quality parameters were estimated (i.e., total sleep time, sleep latency, sleep efficiency, wake time after sleep onset, and awakening index). Validation study was performed prospectively on a separate group of consecutive subjects for whom the breathing analysis was performed using a blind design.

### The experimental system

**Fig 2:**
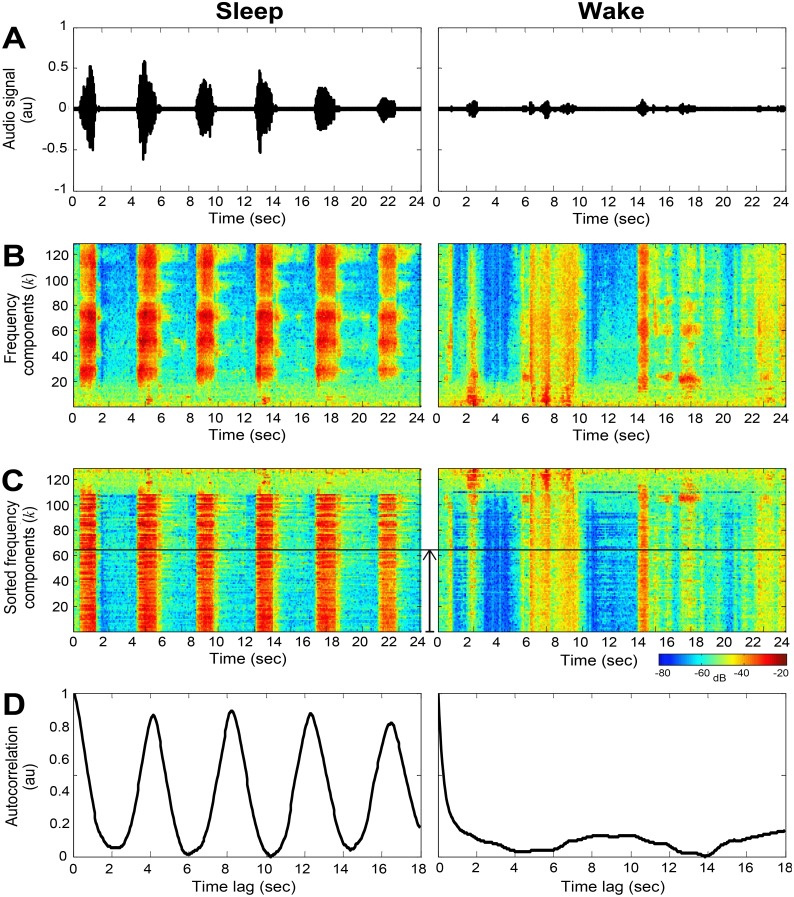
An example of 24-sec interval of audio signal collected from 65-year-old female, BMI 36, AHI 12. Left column illustrates data collected during sleep; right column illustrates data collected during wake. (A) Audio signal following noise suppression. (B) The corresponding spectrogram (frequency components) of the audio signal in (A). (C) The sorted frequency components according to periodicity measure. (D) The enhanced autocorrelation of the interval calculated from the lower half of the sorted frequency components (C), visualized by the vertical solid line.

A digital audio recorder device (Edirol R-4 pro, Bellingham, WA, USA) with a directional microphone (RØDE, NTG-1, Silverwater, NSW, Australia) placed at a distance of 1 meter above the subject's head, was used for acquiring the audio signals. Fig. 2A and E illustrates examples of audio signal (12-sec interval). Data was acquired from a 65-year-old female, body mass index (BMI) of 36 kg/m^2^, apnea-hypopnea index (AHI) of 12 events/hr. The audio signals were stored along with the PSG signals for later analysis. Each audio signal was synchronized with the PSG study at 15 ms resolution according to cross-correlation technique between the PSG (snore intensity level channel) and the digital audio signal (after matching energy sampling rate).

### The sleep-wake pattern estimation algorithm

To estimate sleep/wake activity from audio signal, several steps must be applied. Fig. 1 is the block diagram of the proposed sleep-wake pattern estimation algorithm for the design and validation phases of the study. The algorithm is composed of three basic steps: A) pre-processing, B) feature extraction, and C) sleep-wake pattern estimation. This last step (C) is designed to classify each 30-sec epoch as sleep or awake. It includes classification parameters that were estimated (trained) in the design phase; the training was performed using labeled epochs (sleep and wake) that were derived from the PSG study. The outputs of this algorithm are the whole-night sleep-wake pattern and sleep quality parameters.

#### Preprocessing & noise reduction

For design and validation phases, the acquired audio signals were digitized and stored at 16 kHz, 16 bits per sample. Each audio signal underwent adaptive noise suppression (spectral subtraction) process based on the Wiener-filter. The use of the noise suppression in this system is crucial, since it is designed to emphasize low intensity breathing sounds while suppressing any stationary background noise, such as air-conditioner or fan noises. This process relies on automatically and adaptively tracking background noise segments in order to estimate their spectra and subtracting them from the audio signal [26]. For more information see preprocessing section in the supplements part of Dafna et al [22].

#### Feature extraction

**Fig 3:**
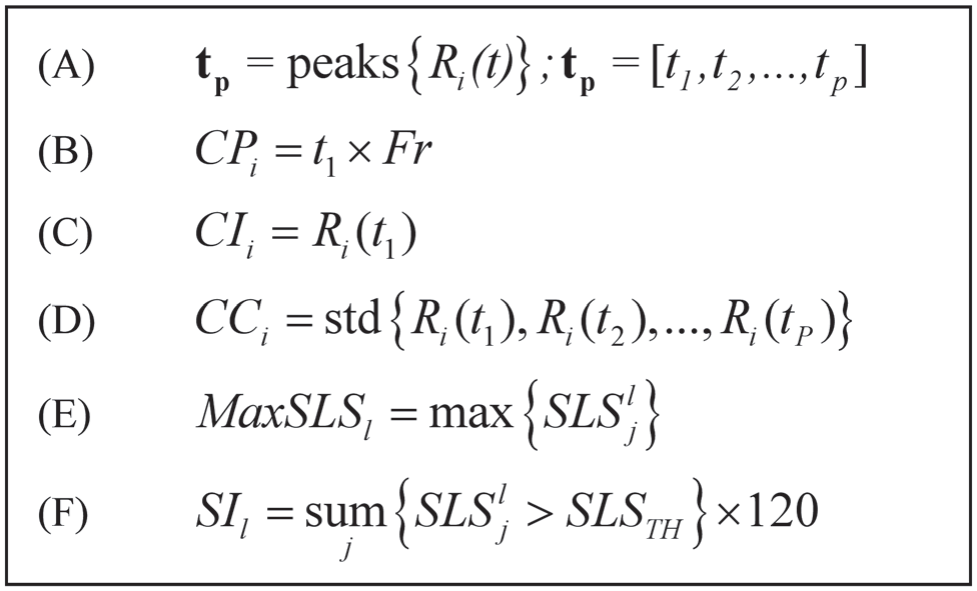
Summary of the equations used for feature extraction process. (A) The function: peaks{·} stands for an operator that finds positive peaks and returns their time-frame index. std{·} stands for standard-deviation function. Index *i* represents the interval of interest (12 or 24 sec), index *j* represents the *j* ^th^ snore index within the *l* ^th^ epoch index. (B) Cycle period feature. (C) Cycle intensity. (D) Cycle consistency. (E) Maximum snore likelihood score in epoch. (F) Snore index.

Eight features were extracted from the full-night audio signal; these features can be divided into 2 categories: A) *breathing pattern*—which is based on periodicity of the energy signal, and B) *Snore properties*—which are based on snore likelihood scores (SLS) [22]. Fig. 3 contains the equations used for the feature extraction process.

*A) Breathing pattern*—these features are designed to capture and quantify breathing pattern such as breathing cycle period, period intensity, and consistency. Since the breathing pattern changes over time, the audio signal is divided into (fixed-length) time intervals. In order to calculate breathing pattern features such as breathing rate, each audio interval should consist of at least two breathing cycles. Therefore we chose an interval length of 12 seconds. In each time interval the autocorrelation of the audio signal is calculated. The autocorrelation function is a mathematical tool for finding repeating patterns; informally, it measures the similarity between signal samples as a function of time lag between them [27]. Since the audio signal may contain noises in different frequencies, the autocorrelation was calculated selectively from part of the spectrum in order to emphasize the periodicity of the interval. For this, a spectrogram, *X*(*k*,*n*), was calculated for each interval (Fig. 2B). The spectrogram of the *i* ^th^ interval, *X*_*i*_(*k*,*n*), presents the running frequency (spectral) components that are calculated from 60-msec signal frames (*k* is the frequency component index and *n* is the time frame index). In order to find repeating breathing patterns, we keep the most periodic information in the spectrogram using the autocorrelation function. Therefore, the autocorrelation is calculated separately for each frequency component:

$$R_{i}(k,t)=\frac{1}{N-t}\sum_{n=1}^{N-t}X_{{}_{i}}(k,n)\times X_{i}(k,n+t),$$ (1)

where *t* is the time-frame-lag index and *N* represents the total number of frames in the interval.

**Fig 4:**
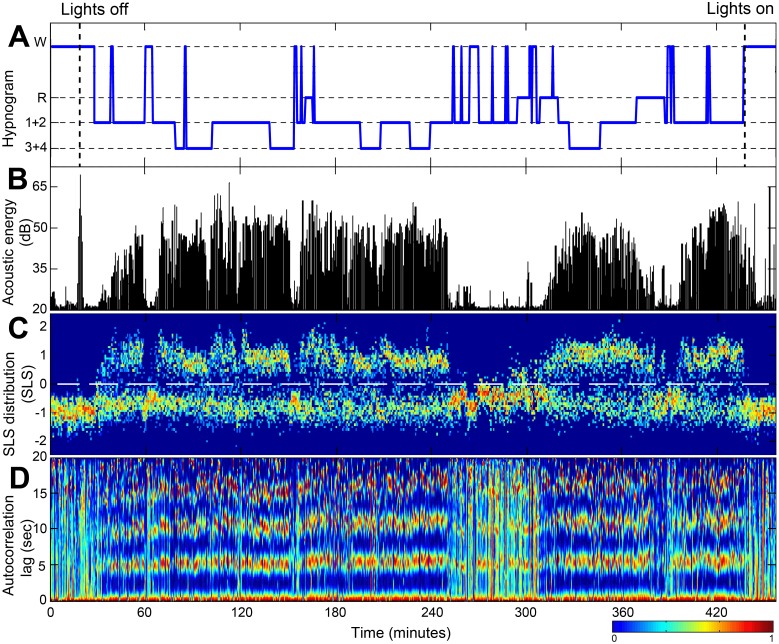
Example of whole night recording and main features. (A) Hypnogram determined by PSG. (B) Audio energy signal after noise reduction. (C) SLS distribution. (D) Autocorrelation of the audio signal over time. Warmer colors represent higher values of SLS distribution (C), and higher values of autocorrelation (D). Note that in (C) during sleep phases there are more SLS values that are above zero (above the horizontal dashed line). In these sleep phases, the autocorrelation (in D) exhibits a more homogeneous stripes pattern compared to the more chaotic behavior in wake phases. For more detailed illustration of the SLS and autocorrelation values during difference vigilance states, see Fig. 5. Data was collected from 52-year-old male, BMI 31. W—wake; R—rapid eye movement (REM); 1+2, 3+4—light and deep non-REM sleep stages, respectively. SLS—snore-likelihood score.

**Fig 5:**
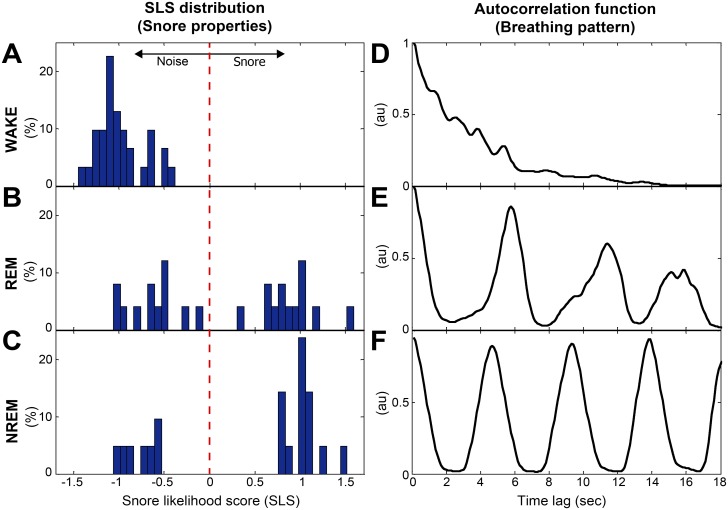
Detailed presentation of the SLS and autocorrelation values during different 30 sec epochs of wake (A, D), REM (B, E), and NREM (C, F). Left panel (A-C): SLS distribution (snore properties), right panel (D-F): autocorrelation function (breathing pattern periodicity). Data is presented from three epochs (wake, REM, NREM) derived from Fig. 4C and D. The dashed line in the left panel represents the decision threshold for snore detection (SLS = 0); higher SLS values indicate greater likelihood of snore event. REM—rapid eye movement, NREM—non-REM.

For each time interval, we calculate an emphasized version of autocorrelation function. The emphasized autocorrelation function, *R*_*i*_(*t*), was calculated as an average function from only the most periodic frequency components according to the *R*_*i*_(*k*,*t*). For that, we sorted the frequency components according to the first peak amplitude value as criterion (see Fig. 3A), and summed the top 50% components (see Fig. 2C). In this stage, the *i* ^th^ interval is represented with a single function of emphasized autocorrelation, *R*_*i*_(*t*) (see Fig. 2D). From every 12 sec time interval, three features are extracted using the emphasized autocorrelation (see equations in Fig. 3): **Cycle period (*CP*)**—which is the location of the first peak, *t*_1_, excluding the zero-lagged peak; **Cycle intensity (*CI*)**—which is the corresponding peak amplitude, *R*_*i*_(*t*_1_); and **Cycle consistency (*CC*)**—which measures how much the breathing pattern is homogenously periodic and consistent within an interval. The more harmonic and repetitive the interval, the lower this feature’s value, and vice versa. The same “breathing pattern” feature extraction process is repeated for 24 sec intervals of the audio signal. Fig. 4 presents an example of the running autocorrelation (used for breathing pattern features extraction) and the running snore properties calculated from a whole-night audio recording. When observing the acoustic energy during sleep (Fig. 4B), breathing becomes more energetic and noisy (sometimes referred to as snores) compared to wake phase. The probability of snores (SLS distribution, Fig. 4C) is also increased during sleep (SLS>0). The running autocorrelation of the breathing pattern, *R*_*i*_(*t*), is shown in Fig. 4D; the running autocorrelation is composed of autocorrelation functions (Fig. 2D) calculated for each epoch (30 sec). Note that sleep episodes exhibit more “zebra-like” stripes patterns compared to the more chaotic one in wake phases, meaning that during sleep, respiration is more periodic, as expected. Fig. 5 illustrates the association between the different vigilance states and the main acoustic features, extracted from specific locations (epochs from Fig. 4C, D). It is clearly notable that there is a similar autocorrelation function during sleep epochs (Fig. 5E, F) in contrast to wakefulness (Fig. 5D). In order to match the *breathing pattern*'s features’ time-resolution (24 sec and 12 sec) to the epoch resolution (i.e., 30 sec, the same as the PSG’s hypnogram), the *breathing pattern* features were linear-interpolated to be sampled at 30 sec resolution.

*B) Snore properties*—these features are based on our previously designed snore detector [22]. Each energetic acoustic event from the audio signal is assigned an SLS that represents its probability of being a snore event; the more positive this score is, the greater its probability to be a snore event. Fig. 4C shows the running SLS distribution (calculated for each 30 sec epoch). Note that in Fig. 4C during sleep phases there are more SLS values that are above zero (above the horizontal dashed line), indicating the presence of snoring events and, consequently, evidence of sleep phase. Fig. 5 left illustrates the association between the different vigilance states and the SLS distribution. It is clearly noticeable that there is a similar SLS histogram during sleep phases (Fig. 5B, C) in contrast to wakefulness (Fig. 5A). Two features were extracted using this SLS parameter: ***Maximum SLS*** in epoch (*MaxSLS*_*l*_) and ***Snore Index*** (*SI*_*i*_). *MaxSLS*_*l*_ feature is calculated as the maximum of SLS within 30-sec epoch *l* (see Fig. 3E). The rationale behind this feature is to determine the epoch's maximum likelihood to contain a snore event. The higher this score is, the more probable this epoch is to contain a sleep episode. The second feature is *snoring index* (*SI*_*i*_) [22], which is the estimation of the number of snores per hour; this estimation is based on counting the snore events within the *l* ^*th*^ epoch and multiplying by the number of epochs per hour; see Fig. 3F for equation details. It is worth noting that each feature was developed to capture breathing sound while minimizing the effect of sound intensity including body posture and distance to microphone [22]. Table 3 summarizes the extracted features.

**Table 3:** Extracted features.

Category	Feature name
Breathing pattern (12 sec)	Cycle period_12_ (*CP^12^*)
	Cycle intensity^12^ (*CI^12^*)
	Cycle consistency^12^ (*CC^12^*)
Breathing pattern (24 sec)	Cycle period^24^ (*CP^24^*)
	Cycle intensity^24^ (*CI^24^*)
	Cycle consistency^24^ (*CC^24^*)
Snore properties (30 sec)	Maximum SLS (*MaxSLS*)
	Snore index (*SI*)

#### Sleep/wake likelihood estimation

**Fig 6:**
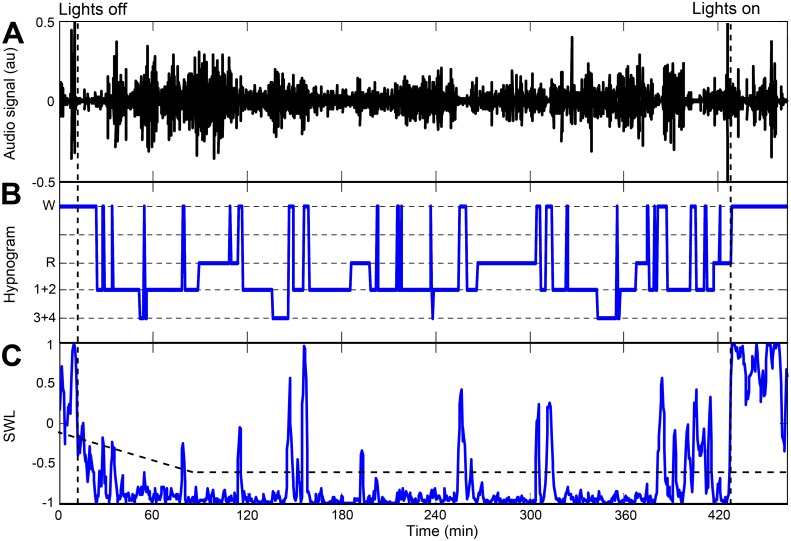
Example of sleep-wake likelihood (SWL) score curve. (A) Audio signal of whole-night recording. (B) Hypnogram. (C) The estimated whole-night SWL score curve. SWL was calculated using the eight acoustic features fed into AdaBoost classifier. Higher values of SWL indicate increased likelihood towards wake state. When focusing on sleep and wake phases, note the similarity between the hypnogram (B) and the SWL (C). The horizontal dashed line represents the corresponding individual decision threshold over time; for more details see main body. Data was collected from 52-year-old male, BMI 31.

Using the eight sleep/wake features, sleep-wake likelihood (SWL) curve is estimated for each patient at 30 second epoch resolution. In this study we chose AdaBoost classifier [25] as a time-series model in which each feature was fed along with two previous epochs (at *l-*1 and *l-*2). In this way, the sleep/wake estimation of the current epoch is supported with the previous epochs' estimations; this approach is suitable for a quasi-stationary process such as sleep and wake phases. Fig. 1B presents a visualization of the time series feature matrix configuration. Generally, a *k*-order AdaBoost classifier involves *k* binary discriminations in a *d*-dimensional feature space (in our case *d* = 24, 8×3), based on the true labeling of the epochs. In the design phase, the classifier parameters were estimated to discriminate two classes: sleep and awake. Sleep was assigned the value '-1' and wake with '+1' (arbitrarily to match the hypnogram), hence producing a linear estimation within that range, i.e., a sleep epoch is more likely to have a negative score than a wake phase. The optimal order was found to be *k* = 100 empirically. Fig. 6 shows a typical example of SWL curve estimated from whole-night audio recording of a subject from the validation design set. Higher values of SWL indicate increased likelihood towards wake state. Note the impressive similarity between the Hypnogram (Fig. 6B) and the proposed acoustic-based SWL curve (Fig. 6C). It should be emphasized that there is a considerable increase in SWL values as soon as wake initiates and it declines immediately during sleep onset.

#### Individual decision threshold

**Fig 7:**
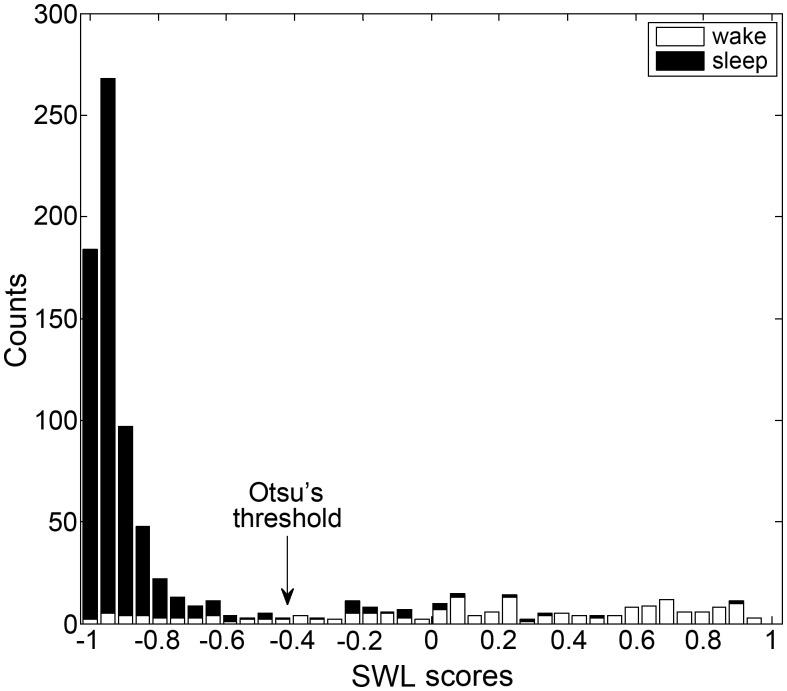
Example of individual decision threshold determination. Stacked-bar-histogram of SWL scores of one subject. Black bars represent sleep epoch scores and white bars represent wake epoch scores. The individual threshold (arrow) was calculated using Otsu's method [28].

Since breathing properties can vary between subjects, we found that applying an individual decision threshold improved system performance. Firstly, for each subject, a likelihood threshold (*L*_*TH*_) was calculated using his SWL epoch scores (example presented in Fig. 6C). These SWL values' distribution (histogram) is considered to behave as a bi-modal distribution, since it is assumed to be composed of sleep and wake phases. The threshold was determined using Otsu’s method [28], which searches for the threshold that minimizes the intra-class variance (see Fig. 7). Secondly, according to our preliminary results, we modified the fixed *L*_*TH*_ to better cope with pinpointing the beginning of sleep, as it may considerably affect estimation of sleep parameters. Therefore, for the first 90 minutes of the recording, the threshold gradually decreases, starting from a higher value towards *L*_*TH*_, in order to reduce false detection of sleep epochs:

$$L_{TH}(t)=L_{TH}+max\{0.5-t/360,0\},$$ (2)

where *t* represents the time index of the epoch, and 360 is a parameter empirically obtained using the design SWL distribution. Note that higher *L*_*TH*_ scores will reduce false detection of sleep episodes. See Fig. 6C for demonstration.

#### Sleep quality parameterization

Using the detected sleep-wake states, we calculated five sleep quality parameters: **1) *Total sleep time*** (*TST*)—actual sleep time in a sleep period; equal to total sleep period less movement and awake time. Total sleep time is the total of all REMS and NREMS in a sleep period. **2) *Sleep latency*** (*SL*)—time period measured from “lights out”, or bedtime, to the beginning of sleep. We measured from start of the recording. **3) *Sleep efficiency*** (*SE*)—The ratio of total sleep time to time in bed. **4) *Wake time after sleep onset*** (*WASO*)—the time spent awake after sleep has been initiated and before final awakening. **5) *Awakening index*** (*AwI*)—the average awakening per hour of time in bed.

#### Data and statistical analyses

**Fig 8:**
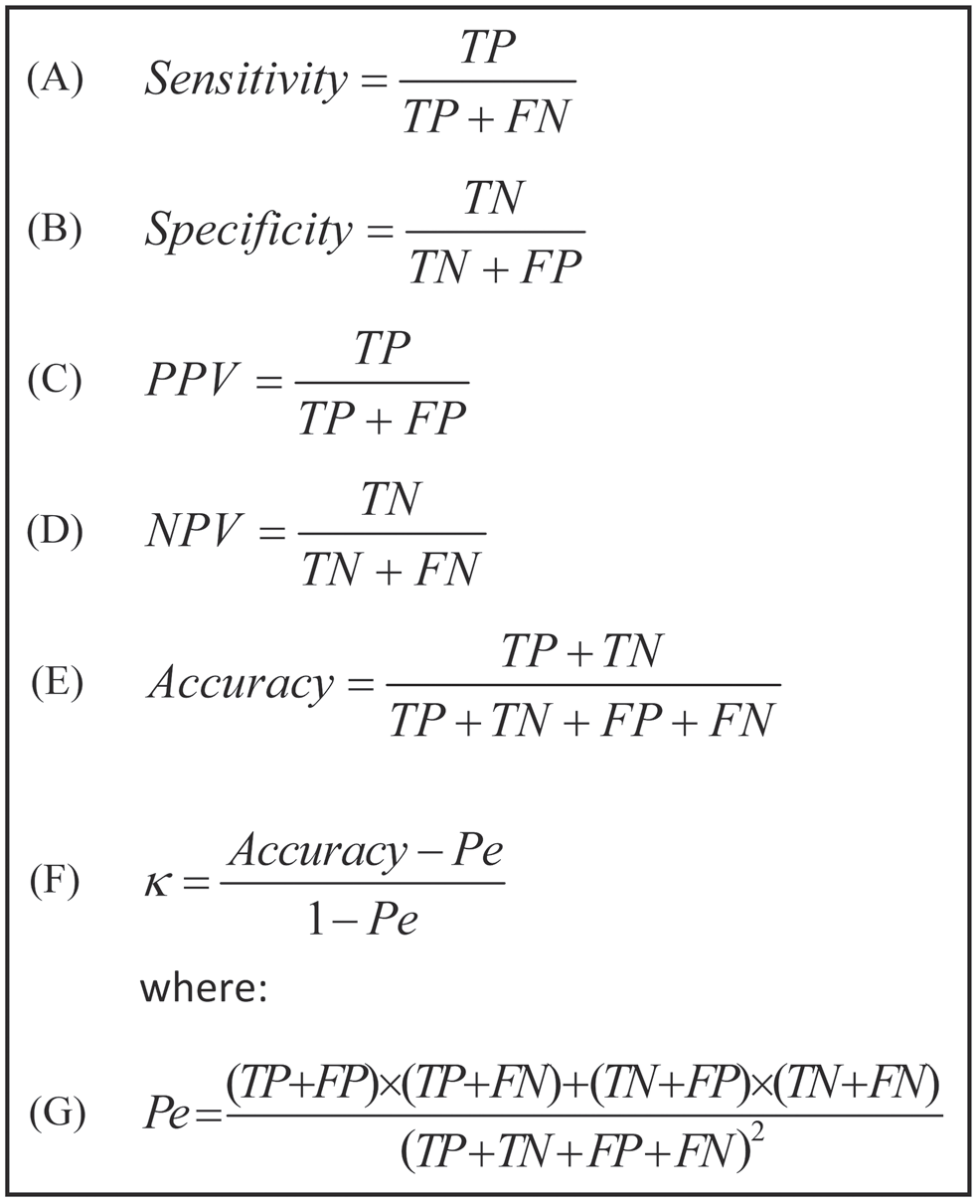
Epoch-by-epoch sleep-wake performance evaluation equations. All of these equations rely on four basic classification parameters: True positive (TP), true negative (TN), false positive (FP), and false negative (FN). We denote 'sleep' as positive and 'wake' as negative. PPV and NPV stand for positive and negative predictive values, respectively. κ—the Cohen's kappa coefficient. *Pe*—probability of agreement by chance.

Audio signal processing and statistical analyses were performed using MATLAB (R-2012b, The MathWorks, Inc., Natick, MA, USA). Both the system design study (*n* = 80) and the validation study (*n* = 70) had similar data handling protocols. A sample size of 64 subjects was calculated to provide a statistical power of 0.80 (*α* = 0.05) in order to achieve >0.45 Cohen's kappa agreement (sleep/wake estimation) for each subject. Therefore, 70 subjects were recruited for the validation study. PSG, demographic, and audio data were compared between design and validation study groups using unpaired two-tailed student *t*-test or χ^2^ test. Epoch-by-epoch sleep-wake estimation performances were calculated using sensitivity (Fig. 8A), specificity (Fig. 8B), positive and negative predictive values (Fig. 8C and D), accuracy (Fig. 8E), and Cohen's kappa (Fig. 8F). We denote “sleep” as positive of interest and “wake” as negative. Hence *TP* (true positive) and *TN* (true negative) represent the counts of true detection of sleep as sleep and wake as wake, respectively. In addition, performances for different working points were obtained from a receiver-operating curve (ROC) and the area under its curve (AUC). Cohen's kappa values are usually associated with five agreement categories [27] (0–0.2 is slight, 0.2–0.4 is fair, 0.4–0.6 is moderate, 0.6–0.8 is substantial, and 0.8–1 is almost perfect). To assess how parameters such as age, gender, BMI, AHI, and signal-to-noise-ratio (SNR) affected the kappa agreement of our BSA system, we tested the person correlation coefficient for each of these parameters.

In the analyses, first, for each subject in the validation dataset, we performed epoch detection performances as described above. A global average and standard deviation were calculated among all validation subjects. Second, based on the estimated epochs, for each subject, we calculated the five sleep quality parameters and compared to PSG. Then a global average and standard deviation were calculated among subjects. For better visualization of system performance, we presented each subject's sleep quality parameter using Bland-Altman plot method [29].

## Results

### Subjects and PSG characteristics

One hundred and fifty subjects referred to PSG evaluation of sleep disorders were included in our study (Table 1). An average of 7.1±0.9 hours of audio signals were recorded from each subject with no significant differences between design and validation studies; a total of 562.9 hours and 499.9 hours were analyzed in the design and validation studies, respectively (Table 2). No significant differences in subject anthropometric parameters (Table 1) and sleep quality parameters (Table 2) were found between system design and validation study groups. As a group, both design and validation patients have moderate obstructive sleep apnea and fragmented sleep. In this study we included 127,668 manually examined epochs: 59,108 design study and 68,560 validation study. Figure A in S1 Dataset illustrates individual big-data visualization for the design study (n = 80) and validation study (n = 70).

### Feature extraction

Eight acoustic features were developed in this study (Table 3). These features were calculated at different interval durations in order to capture breathing sounds and to extract sleep-wake pattern information. These features are based on short- and long-term analyses, and later were adjusted to construct a time-series feature matrix at a 30 sec epoch resolution. Fig. 6D shows an example of sleep-wake prediction according to each of the developed features from a 52-year-old male, AHI 22 events/hr, BMI 31 kg/m^2^. Note that each feature exhibits the actual sleep-wake pattern determined by PSG.

**Fig 9:**
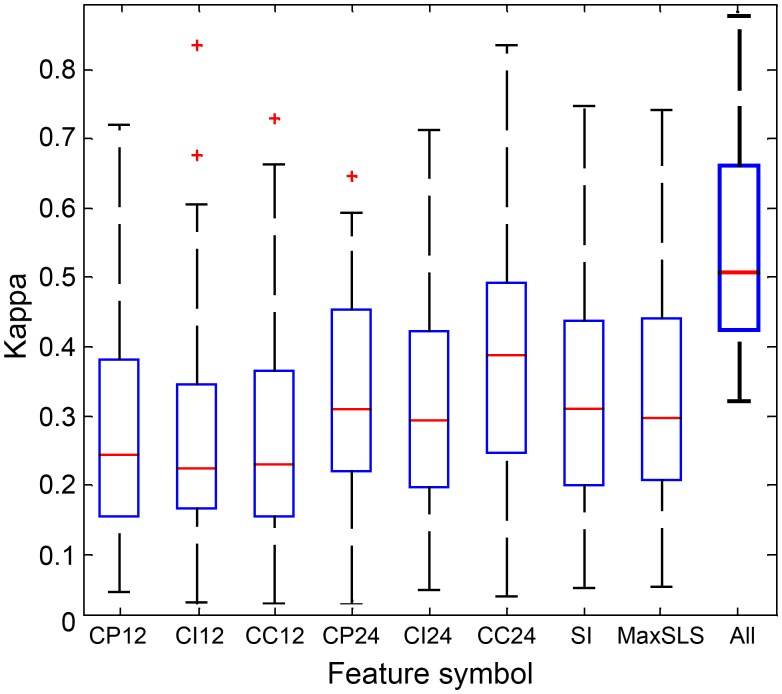
Feature performances. Boxplot representing the distribution of kappa agreement values for all subjects within the validation dataset using Otsu's threshold [28]. The rightmost box represents the agreement derived using all features as a group (using the AdaBoost classifier).

Fig. 9 presents evaluation (kappa values) of each individual feature to distinguish between sleep and wake epochs using the study validation dataset. Each feature is presented as a boxplot, measuring the quartile distribution of kappa agreements of sleep-wake epochs among various patients. Note the relatively wide range of kappa values, indicating the large variability between subjects. The median kappa agreement of each feature is between 0.2 and 0.4. However, when combining all features, the overall kappa agreement is improved to a narrower distribution, i.e., median kappa of 0.5 and quartiles of 0.4 to 0.7.

### Sleep/wake pattern estimation

During the design phase, an AdaBoost model was trained on the entire design dataset in order to classify sleep and wake epochs. Based on the SWL score for each subject, an individual threshold was picked to better fit the global sleep-wake model to the individual. Each subject’s scores (for sleep and awake epochs) were shifted using this threshold value, allowing adaptation for the global sleep-wake model to better fit the individual subject. Moreover, global alignment of the scores from all the subjects will allow standardization and overall evaluation of system performance. On average, the individual *L*_*TH*_ improved system accuracy by 3%. Fig. 6C shows a typical example of the SWL curve of an individual; the horizontal dashed line represents the calculated *L*_*TH*_ score.

**Fig 10:**
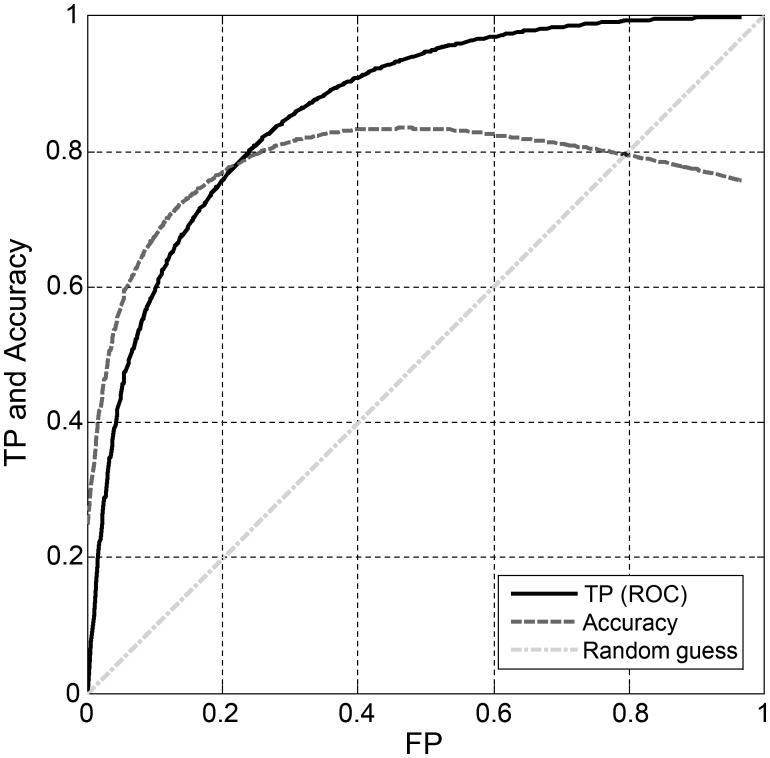
Receiver operating characteristics (ROC) plot of epoch-by-epoch sleep/wake estimation system performances. Solid line represents the TP of the system as a function of FP, the dashed line represents the accuracy as a function of FP. Dot-dashed line represents the random-guess performance. TP—True positive of detecting sleep epochs. FP—false positive.

Epoch-by-epoch system performances (sleep/wake estimation) were analyzed for each subject (individual performances). Based on the individual performances, we calculated a global mean and standard deviation between subjects. Table 4 shows the comparison between the proposed BSA and PSG for the entire group of subjects using the validation dataset. The overall (global) accuracy between subjects of the BSA system was 0.833. The sensitivity (detecting sleep epochs as sleep epochs) was 0.922 and the specificity was 0.566. Cohen's kappa agreement was 0.508. The ROC curve (Fig. 10, solid line) has an area under the curve of 0.829. Accuracy, sensitivity, specificity, positive and negative predictive values, area under the curve, and Cohen's kappa were also calculated for the entire pool of validation epochs (regardless of subjects’ epoch origin), and no discernable differences were observed between the two cases (data not shown). To assess how parameters such as age, gender, BMI, AHI, and SNR affected the kappa agreement of our breathing sound analysis (BSA) system, we tested the Pearson correlation coefficient for each of these parameters. Our calculations reveal negligible correlations (<0.1) for age, gender, BMI, and AHI. Correlation between system performances and breathing SNR reveals positive correlation of 0.37 (*p*<.001) (Figure B in S1 Dataset).

**Table 4:** Performance of BSA vs. PSG classification (epoch-by-epoch).

**Performance**	**SEN**	**SPC**	**PPV**	**NPV**	**ACU**	**AUC**	**kappa**
Subject's Mean	.922	.566	.859	.723	.833	.829	.508
Subject's standard deviation	.078	.179	.102	.184	.076	.065	.156

SEN = sensitivity; SPC = Specificity; PPV = Positive predictive value; NPV = negative predictive value; ACU = accuracy; AUC = Area under ROC curve; kappa = Cohen's kappa coefficient. The mean and standard deviation values represent the distributions of subject's performances (validation dataset).

### Sleep quality parameterization

**Fig 11:**
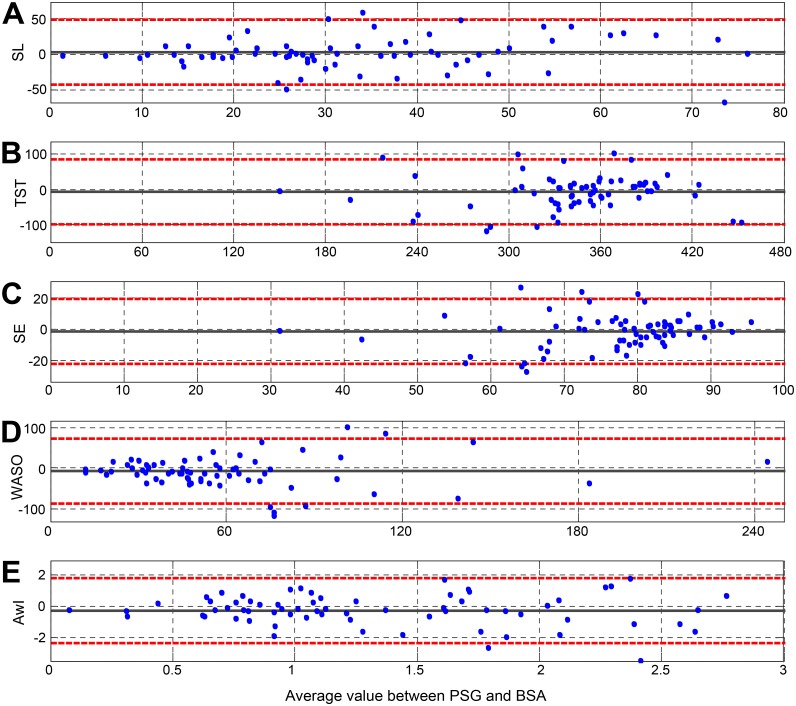
Bland-Altman plot of the PSG & BSA. X-axis represents the mean sleep parameter value between the PSG & BSA (in the relevant parameter units). The Y-axis is the difference between the PSG and BSA sleep quality parameter. The dashed lines represent the 95% CI. (A) SL—Sleep latency, (B) TST—Total sleep time, (C) SE—Sleep efficiency, (D) WASO—Wake time after sleep onset, (E) AwI—the average awakening per hour of time in bed.

No significant differences in sleep quality parameters as measured by PSG and BSA were found (Table 2). The differences between the sleep quality parameters as measured by PSG and BSA are presented to show the direction of any bias. The average difference between SPG and BSA were: TST (7.6±48.1 min), SL (-3.3±23.5 min), SE (1.5±10.7%), WASO (7.8±41.3 min), and AwI (0.2±1.0 per hr). Moreover, the absolute error (differences) was presented to quantify the overall magnitude of differences among measurements. The absolute error between SPG and BSA were: TST (35.8±32.8 min), SL (16.6±16.9 min), SE (8.0±7.3%), WASO (29.6±29.7 min), and AwI (0.8±0.8 per hr). Fig. 11A-E shows evaluation of five sleep quality parameters: TST, SL, SE, WASO, and AwI, according to the Bland-Altman plot. Examining the Bland-Altman plots and comparing the proposed breathing sound analysis (BSA) approach versus PSG, we found good agreement. All sleep quality parameters show no major consistent bias (Fig. 11 and Table 2). The plots also show (for SL, WASO, and AI) that the BSA values are closely matched to PSG in the left part of the plot, and therefore present less difference when the parameter values are relatively small; this implies reliability of the estimated parameters.

## Discussion

In this paper a novel method for evaluation of sleep-wake patterns using non-contact microphone is proposed. This method utilizes analysis of breathing sounds and machine-learning techniques in order to reliably estimate sleep-wake activity and to reliably estimate sleep quality parameters. The main idea beyond this approach is that central control of ventilation and upper airway patency are strongly affected by sleep and wake activity [4,15]. The human upper airway is a complicated structure comprising a number of muscles whose functional integration is essential for several complex tasks including speech [30] and breathing [20]. From the respiratory perspective, the primary goal of these pharyngeal muscles is to keep the airway open. While the pharyngeal muscles manage this task with relative ease during wakefulness, their activity is often diminished at sleep onset [19,31,32]. During sleep, there is a considerable increase of upper airway resistance [4,17,18,33] due to decreased activity of the pharyngeal dilator muscles [19,20]. This elevated resistance is reflected by amplification of air-pressure oscillations in the upper airways during breathing. These air-pressure oscillations are perceived as typical breathing sounds during sleep. In contrast, during wakefulness, there is an increase in activity of the upper airway dilating muscles, hence decreased upper airway resistance [19] and airway oscillations.

This study provides evidence that by audio analysis of breathing sounds, the breathing pattern and hence the sleep-wake activity can be estimated. We developed and validated a system that incorporates eight features (extracted mainly from the audio energy signal; see Methods) that were designed to measure and quantify breathing pattern and breathing properties. These features were used for training an AdaBoost [25] classification model to estimate sleep and wake activity at 30 sec epoch resolution. Using the estimated epoch labels (sleep/wake), we calculated five acceptable sleep quality parameters. System performances were analyzed epoch-by-epoch. The accuracy was 0.833, the sensitivity was 0.922, and the specificity was 0.566. Cohen's kappa agreement was 0.508. When analyzing sleep quality parameters (BSA vs. PSG), the average error (in minutes) of sleep-latency, total sleep time, and wake after sleep onset was 16.6, 35.8, and 29.6, respectively.

### Breathing signals and analysis

Extracting sleep-wake activity pattern from breathing sounds using a non-contact microphone is challenging. Since breathing sounds may be “contaminated” by background noise, it is essential to improve SNR prior to analysis in order to enhance the breathing events. To achieve this, we used an adaptive spectral subtraction technique [26] that subtracts the estimated background noise. This technique is acceptable in the speech and audio signal enhancement field [34]; however, it was not fully explored on whole-night breathing sounds. By applying this technique, background noise was considerably reduced, similar to earlier studies [22,34].

We developed a unique set of features that are specifically designed to distinguish between sleep and wake phases (Table 3, Fig. 4). These features can be categorized into two categories: *breathing pattern* and *snore-characteristics*. The *breathing pattern* features are designed to capture and to quantify variations in breathing periodicity as they may contribute to distinguishing between sleep and wake epochs. The basic idea here is that the new developed features: CP, CC, and CI (collectively named “periodicity features”; see Methods) are strongly influenced by breathing properties, such as rate and consistency. It was well established that changes in vigilance states strongly affect breathing rate and regularity in humans and animals [4,16,19,33,35–39]. The *snore-characteristics* features are designed to find the probability of a given epoch to contain snores. The basic idea is that the probability of detecting snoring events is increased during sleep. Snoring is caused by the vibration of soft tissue in the upper airways due to elevated upper airway resistance during sleep [21]. Recently, it was shown that snore analysis may carry valuable information about sleep conditions [23,40]. In order to calculate these *snore-characteristics* features, we used our high performance (>98% detection accuracy rate) snore detector [22] module. By using all these (eight) features as a multi-dimensional input to our sleep-wake classifier, the performances were superior to using each feature separately (Fig. 9).

We used the AdaBoost [25] algorithm to classify epochs as sleep or wake. The main advantage of this algorithm is its ability to discriminate multi-dimensional complex-patterns using a non-parametric, non-linear boundary threshold [25]. The use of this kind of classifier was supported by an earlier study [41], which claimed that sleep and wake activity (using wrist-actigraph) should be discriminated using non-linear classifiers. In addition, other studies have shown that there is a strong correlation between the labels (sleep/wake) of adjacent (30 sec) epochs [28,42]. Therefore, we configured our AdaBoost classifier as a time-series model, in which the prediction of each epoch state is influenced by the adjacent epochs, i.e., it is unlikely (though it's possible) to find a fragmented sequence of [wake-sleep-wake] and vice versa. In our earlier study [43] we also explored the effect of different classifiers such as two states hidden Markov models [44]. The time-series AdaBoost classifier was found to produce the best results and therefore was chosen.

### Comparison to existing approaches

Little information is available about sleep evaluation using a non-contact audio-based approach. In recent years, several other simple cost-effective technologies for sleep evaluation have been presented. These technologies are based on reduced-channels and sensors, in which the preferred methods to detect sleep and wake phases are based on detecting patient movement. The most validated approach is actigraph [5,45]. Hedner et al. [3], Marino et al. [11], Lotjonen et al. [46], and Tilmanne et al. [41] have validated the performance of actigraphy versus PSG using in-laboratory data collection. They showed an agreement (accuracy) ranging between 80% and 86%. Innovations in mobile and electronic healthcare are revolutionizing the involvement of both doctors and patients in the modern healthcare system by extending the capabilities of physiological monitoring devices. Incorporating smart wearable sensors into the routine care of patients could augment physician-patient relationships, and increase the autonomy and involvement of patients in their healthcare [12]. However, such systems are rarely tested scientifically [13]. Our BSA system yields matched and even superior performances relative to an actigraph technology. Furthermore, in our system, according to Bland-Altman plot (Fig. 11), all sleep quality parameters showed no consistent bias, where actigraphy-based technology showed some bias in the sleep quality parameters [11,47,48]. When calculating the average error (mean absolute difference) of the sleep quality parameters between PSG and actigraph, Lotjonen at el. [46] and Blackwell et al. [49] showed a TST estimation error of 54 min and 44 min, respectively. Blackwell et al. [49] also calculated the estimation error of SE and WASO; they showed an average error of 9.8% for sleep efficiency and 39 min for WASO. By comparing the errors of these sleep quality parameters, our BSA system yields superior performances (TST 35.8 min, SE 8%, and WASO 29.6 min).

In addition to actigraphy-based methods, some researchers took movement detection even further in order to estimate sleep and wake activity in non-contact technologies [50–52]. Collectively, these studies showed performances that are inferior to or close to actigraphy-based approaches. It is generally recognized that body-movement-based technologies (contact and non-contact) are biased [9,11,47,48], since any lack of movement is interpreted as sleep, while movement is interpreted as awake. In contrast, our approach of estimating sleep-wake from breathing sounds is based on the notion that during sleep onset there are considerable changes in upper airway mechanics, affecting airflow and pressure oscillations. In contrast to these movement-based technologies, we found that our system performance is not influenced by any physiological variables, such as age, BMI, AHI, and gender.

### Strengths and limitations

We provide evidence that sleep-wake activity can be reliably estimated by audio analysis of breathing sounds. In this study 127,668 epochs were analyzed from 150 subjects who were referred to sleep evaluation; these subjects represent a diverse population, across a wide range of ages, BMI, AHI, and from both genders, but are not strictly a generalizable population. Our proposed non-contact technology may enable more natural sleep that is not affected by the equipment. It is expected that the wear and tear and costs will be relatively low; however, further studies should determine whether this technology is valid in at-home conditions and its cost-effectiveness. Clearly, the transition of this technology to at-home sleep evaluation mainly depends on third party reimbursements for the use of home study equipment [11,53]. It should be recognized that the main limitation of this technology is its sensitivity to low SNR, which results from quiet breathing relative to high environmental background noise. In addition, at the current state of operation, our system is limited to operate in a single-subject environment (one person in the room); since other person's breathing sounds can interrupt. Further studies should investigate methods to cope with several subjects in the room and to further improve SNR mechanically and algorithmically.

## Summary

One of the main goals of sleep medicine today is to improve early diagnosis and treatment of the “flood” of subjects presenting with sleep disorders. New simple technologies are needed in order to improve patient accessibility to sleep diagnosis; this in turn will reduce the cost of management and treatment [53], and improve quality of life and health.

This study presents a pioneering approach for determining sleep-wake pattern using non-contact audio-based signals. We found that by analyzing breathing sounds a reliable estimation of sleep quality parameters can be achieved. This study highlights the potential of breathing sound analysis to measure sleep in research and clinical situations.

## Supporting Information

S1 Dataset This file contains Figure A and Figure B. Figure A, Individual big-data visualization for the study design (training, n = 80) and validation (testing, n = 70): design dataset—upper panels; validation dataset—lower panels. Each horizontal line represents one individual’s data. Sleep/wake activity pattern was manually annotated epoch-by-epoch using polysomnography (PSG) scoring criteria. Note the large individual differences in sleep-wake pattern, especially the sleep latency (from time zero to the first black mark of sleep) and the large individual variability of awakening during sleep (mustard colors between black regions). The onset of the gray area indicates study termination for each individual. Cohen's kappa (epoch-by-epoch sleep/wake) agreement score for each subject was calculated comparing our proposed breathing sound analysis (BSA) system and the PSG scoring. For study protocol, see main body of the manuscript. Figure B, The correlation between sleep-wake estimation performances (using kappa score) and subject's breathing sound recording quality (signal to noise ratio, SNR). Each dot represents the mean SNR of one individual from the validation (testing) dataset (n = 70).
(PDF)
