# Supplementary material for: Sleep-Wake Evaluation from Whole-Night Non-Contact Audio Recordings of Breathing Sounds
Source: PLoS One. 2015 Feb 24;10(2):e0117382. doi: 10.1371/journal.pone.0117382 (PMC4339734; doi:10.1371/journal.pone.0117382)
Supplement: S1 Dataset — Figure A, Individual big-data visualization for the study design (training, n = 80) and validation (testing, n = 70): design dataset—upper panels; validation dataset—lower panels. Each horizontal line represents one individual’s data. Sleep/wake activity pattern was manually annotated epoch-by-epoch using polysomnography (PSG) scoring criteria. Note the large individual differences in sleep-wake pattern, especially the sleep latency (from time zero to the first black mark of sleep) and the large individual variability of awakening during sleep (mustard colors between black regions). The onset of the gray area indicates study termination for each individual. Cohen's kappa (epoch-by-epoch sleep/wake) agreement score for each subject was calculated comparing our proposed breathing sound analysis (BSA) system and the PSG scoring. For study protocol, see main body of the manuscript. Figure B, The correlation between sleep-wake estimation performances (using kappa score) and subject's breathing sound recording quality (signal to noise ratio, SNR). Each dot represents the mean SNR of one individual from the validation (testing) dataset (n = 70). (PDF) [file pone.0117382.s001.pdf]

ONLINE SUPPORTING INFORMATION S1 DATASET

Sleep-wake evaluation from whole-night non-contact audio recordings of breathing sounds

Eliran Dafna, Ariel Tarasiuk, Yaniv Zigel

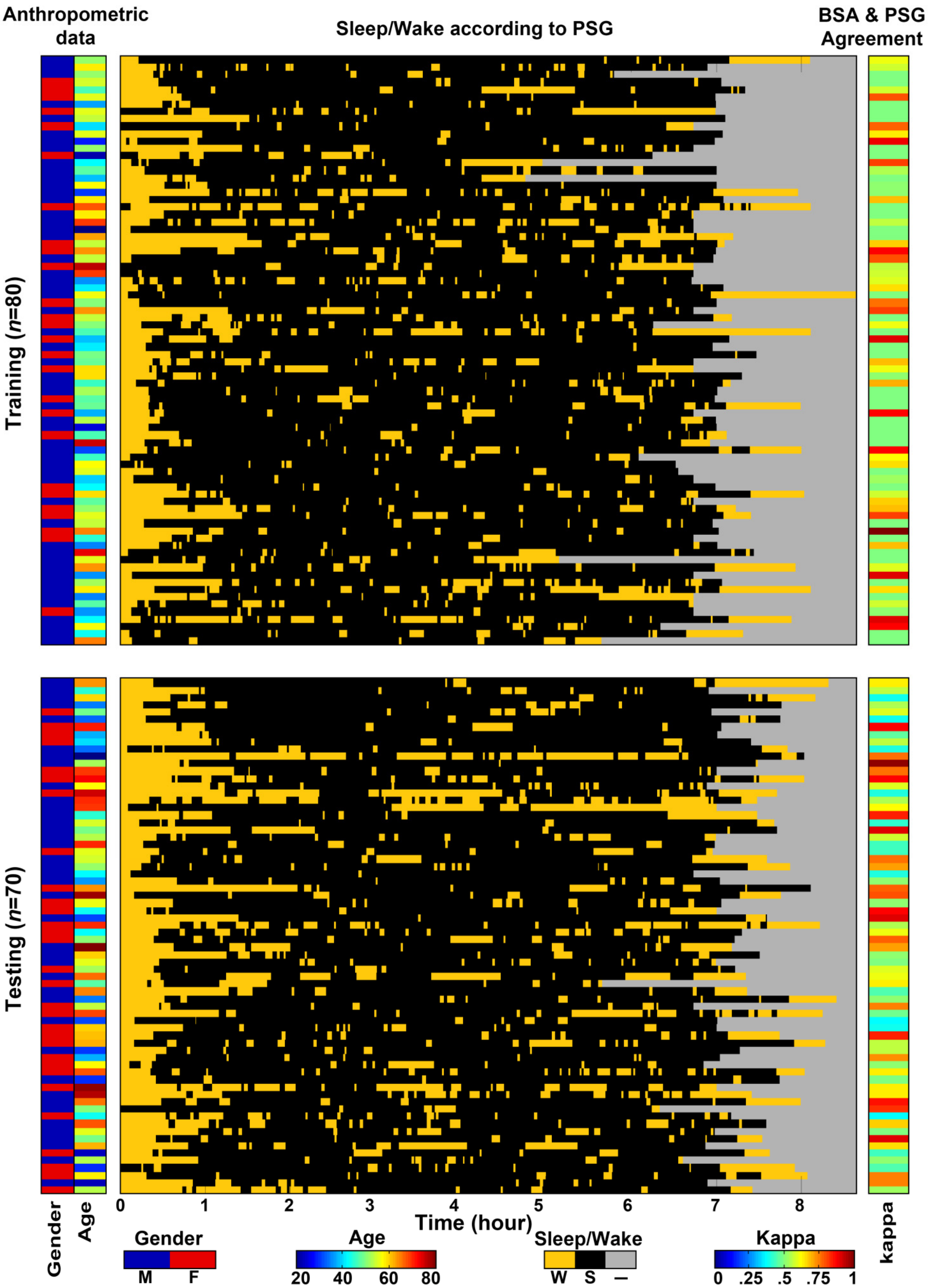

Figure A – Individual big-data visualization for the study design (training, *n*=80) and validation (testing, *n*=70): design dataset – upper panels; validation dataset – lower panels. Each horizontal line represents one individual's data. Sleep/wake activity pattern was manually annotated epoch-by-epoch using polysomnography (PSG) scoring criteria. Note the large individual differences in sleep-wake pattern, especially the sleep latency (from time zero to the first black mark of sleep) and the large individual variability of awakening during sleep (mustard colors between black regions). The onset of the gray area indicates study termination for each individual. Cohen's kappa (epoch-by-epoch sleep/wake) agreement score for each subject was calculated comparing our proposed breathing sound analysis (BSA) system and the PSG scoring. For study protocol, see main body of the manuscript.

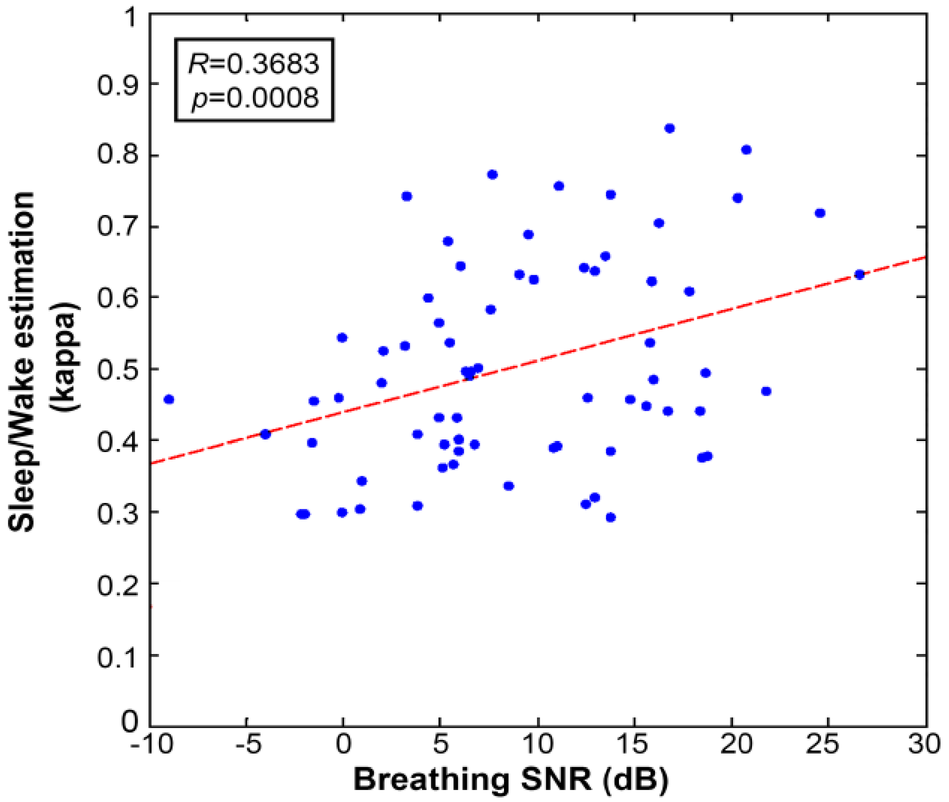

Figure B – The correlation between sleep-wake estimation performances (using kappa score) and subject's breathing sound recording quality (signal to noise ratio, SNR). Each dot represents one individual from the validation (testing) dataset (*n*=70).
